# A Review on the Application of Zeolites and Mesoporous Silica Materials in the Removal of Non-Steroidal Anti-Inflammatory Drugs and Antibiotics from Water

**DOI:** 10.3390/ma14174994

**Published:** 2021-09-01

**Authors:** Agnieszka Grela, Joanna Kuc, Tomasz Bajda

**Affiliations:** 1 Faculty of Environmental and Power Engineering, The Cracow University of Technology, 30-155 Cracow, Poland; 2 Faculty of Geology, Geophysics and Environmental Protection, AGH University of Science and Technology, 30-059 Cracow, Poland; jkuc@agh.edu.pl (J.K.); bajda@agh.edu.pl (T.B.); 3 Faculty of Chemical Engineering and Technology, The Cracow University of Technology, 30-155 Cracow, Poland

**Keywords:** zeolites, mesoporous sorbents, wastewater purification, drug analysis

## Abstract

Zeolites and mesoporous silica materials are effective adsorbents that can be useful for the removal of various pharmaceuticals including non-steroidal anti-inflammatory drugs and antibiotics from low-quality water. This paper summarizes the properties and basic characteristics of zeolites and mesoporous silica materials and reviews the recent studies on the efficacy of the adsorption of selected non-steroidal medicinal products and antibiotics by these adsorbents to assess the potential opportunities and challenges of using them in water treatment. It was found that the adsorption capacity of sorbents with high silica content is related to their surface hydrophobicity (hydrophilicity) and structural features, such as micropore volume and pore size, as well as the properties of the studied medicinal products. This review can be of help to scientists to develop an effective strategy for reducing the amount of these two groups of pharmaceuticals in wastewater.

## 1. Introduction

Pharmaceuticals are substances with a high biological activity, which are introduced into the body in a strictly defined dose to achieve a desired (therapeutic or preventive) effect. They are exposed to the environment in many ways, the most important being excretion by humans and animals and inappropriate disposal of drugs. These compounds are excreted from the body in the form of parent compounds or as metabolites formed in the first and second phase of biotransformation [1,2,3]. Many pharmaceutically active compounds were already detected in water in the 1980s. Bush (1997) grouped these therapeutic substances into the following classes: (a) anti-inflammatory agents and analgesics, (b) antibiotics, (c) antiepileptics, (d) antidepressants, (e) lipid-lowering agents, (f) antihistamines, (g) β-blockers, and (h) other substances [3,4].

**Figure 1:**
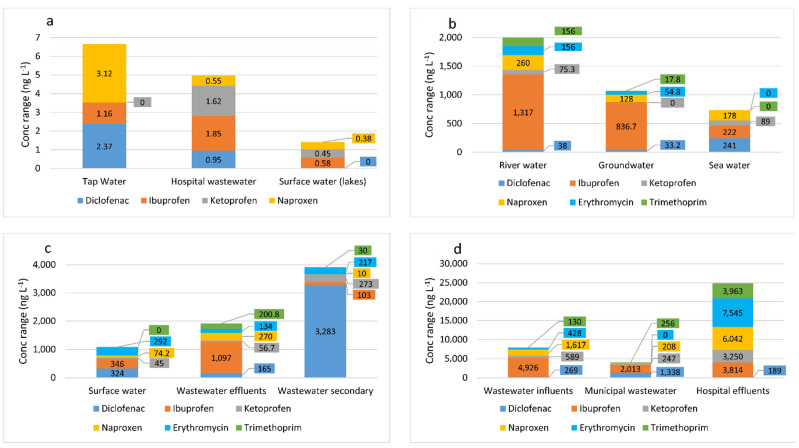
Concentrations of some pharmaceuticals (antibiotics, anti-inflammatory drugs, and analgesics) recorded in the world in selected water types: (**a**) tap water [25], hospital wastewater, and surface water (lakes) [26]; (**b**) river water, groundwater [27], and seawater [28]; (**c**) surface water [29], wastewater effluents, and wastewater secondary [27,30]; and (**d**) wastewater influents [30], municipal wastewater [29], and hospital effluents [30].

Pharmaceuticals that are most frequently detected, including antibiotics, anti-inflammatory drugs, and analgesics, have become a growing environmental concern worldwide [5,6]. They occur mainly in the aquatic environments, such as surface and underground waters, water reservoirs, effluents and influents of sewage treatment plants, and drinking water [7,8,9,10,11,12,13]. Medicines are found in trace concentrations up to 100 µg L^−1^ in wastewater resulting from drug production [14]. Drugs are found in the environment because these pollutants cannot be completely removed in sewage treatment plants [15], and thus persist without undergoing degradation [16]. Incomplete elimination of pharmaceuticals was also observed in drinking water treatment plants [17,18]. This paper focuses on two groups of pharmaceuticals—non-steroidal anti-inflammatory drugs (NSAIDs) and antibiotics—as they are the most widely used medicinal products worldwide. NSAIDs (diclofenac, ibuprofen, ketoprofen, and naproxen) were chosen owing to their large-scale use and widespread distribution in surface waters and wastewater, which is confirmed by numerous scientific studies [19,20,21]. In turn, the antibiotics discussed in this article (erythromycin, sulfamethoxazole, tetracycline, and trimethoprim) were selected as they are included in the World Health Organization’s List of Essential Medicines [22] and are also used widely as antimicrobial substances against bacteria [20,23,24]. Of the NSAIDs of interest, only ibuprofen is on the WHO list. Figure 1a–d presents the concentrations of selected drugs from the group of NSAIDs and antibiotics found in the aquatic environment based on data from the analyzed studies.

Based on the data from Figure 1a–d, it can be stated that antibiotic concentrations were highest in hospital effluents, wastewater effluents, and river water. In three types of water (tap water, hospital wastewater, surface water (lakes)) presented in Figure 1a, erythromycin and trimethoprim were not detected. The highest concentrations of diclofenac, ibuprofen, and ketoprofen were found in wastewater influents, municipal wastewater, and hospital effluents. Thus, it can be concluded that the drug concentrations in different types of waters and wastewater are found in the following order: hospital effluents > wastewater influents > municipal wastewater > secondary wastewater > river water > wastewater effluents > groundwater > surface water > seawater > tap water > hospital wastewater > surface water (lakes). Owing to human activity, pharmaceuticals are detected in various types of water and wastewater on each continent, including the North Scandinavian water environment [31]. The strategy for their removal can be the same everywhere, as long as the concentrations are at a similar level. The most easily removable drugs are mainly those belonging to the group of non-steroidal analgesics and anti-inflammatory drugs, including ibuprofen, ketoprofen, and naproxen. On the other hand, the elimination of pharmaceuticals such as diclofenac from wastewater is difficult.

**Figure 2:**
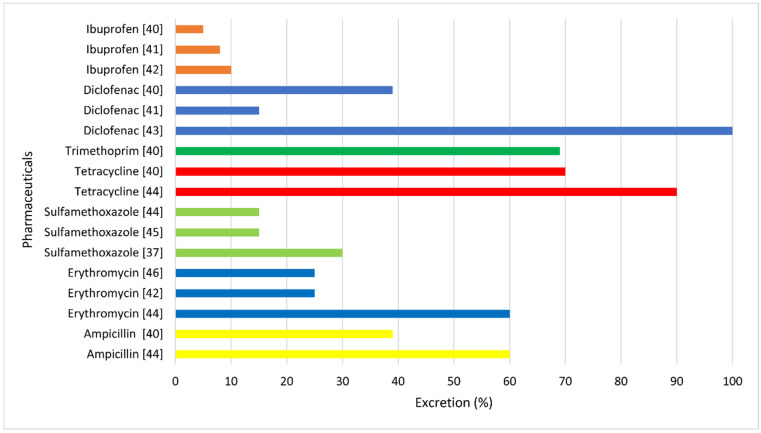
Typical pharmaceuticals and their approximate nonmetabolized fractions entering sewage after being ingested and subjected to human metabolism: ibuprofen [40,41,42]; diclofenac [40,41,43]; trimethoprim [40]; tetracycline [40,44]; sulfamethoxazole [37,44,45]; erythromycin [42,46]; and amplicillin [40,44].

Pharmaceuticals are chemically stable. However, owing to physicochemical and biotic factors [32], they undergo biodegradation, conjugation, deconstruction, and sorption. Therefore, the knowledge of these processes is necessary to predict the environmental fate of medicinal substances [13]. The high stability of drugs is related to their relatively high durability under environmental conditions. In contrast, some pharmaceutical metabolites resulting from oxidation, reduction, and/or hydrolysis are more susceptible to further transformations, and thus are less stable in the aquatic environment [33]. The transformations of pharmaceuticals taking place in the aquatic environment are not thoroughly studied so far [34,35,36]. Pharmaceuticals undergo many reactions and changes, the first of which dilutes the drugs when they reach the surface water and water reservoirs [37], while chemical reactions may partially or completely change the original pharmaceuticals (parent compounds) [1]. The products resulting from the transformation of pharmaceutical compounds are sometimes more stable than the parent compounds and may be more or less toxic. Moreover, pharmaceuticals may undergo biotic (aerobic and anaerobic) and abiotic (chemical) reactions in the environment [15,38]. Most often, pharmaceuticals are trapped in sewage sludge, but their original molecular structures are preserved. This is generally observed in the case of lipophilic and difficult-to-degrade substances. Pharmaceuticals also possibly transform into hydrophilic compounds, which remain stable. Such hydrophilic products pass through sewage treatment plants and reach the flowing surface waters (rivers) and still surface waters (water reservoirs and lakes) [5]. It has been shown that pharmaceuticals exhibit a very wide range of removal rates without any logical scheme, even if they belong to the same therapeutic groups [39]. Figure 2 presents the approximate nonmetabolized fractions of selected pharmaceuticals from the NSAID group and that of antibiotics entering wastewater after ingestion and human metabolism. The *x*-axis excretion percentage represents unmetabolized or partially metabolized pharmaceuticals that are eliminated as the original active ingredient.

Pharmaceuticals enter the environment mainly by water transport and further spread into the environment through the food chain [7]. The side effects of these substances are still unknown and have not been tested. Pharmaceuticals can affect aquatic ecosystems, but the extent of this damage is not clear [5,47]. Some studies have already reported that these compounds pose both acute and chronic threats to flora and fauna. It has been proven that diclofenac has a negative effect on vultures, causing a decline in their population [48]. In turn, Schwaiger et al. [49] and Triebskorn et al. [50] indicated that exposure of rainbow trout to diclofenac results in damage to internal organs. Sulfamethaxazole has also been shown to affect the germination of rice and oats [51].

Because of the above-described consequences, it is necessary to optimize and improve the technologies currently used for the treatment of wastewater and surface water in order to eliminate pharmaceutical residues from them. Because the biological and physical removal efficiency of these residues is not very high, there is a need to search for other more effective cleaning methods. Chemical (e.g., ozonation and oxidation) and physicochemical processes (e.g., adsorption, membrane filtration, and coagulation) are commonly used for the removal of medicines from aqueous solutions [52,53,54]. Some of the pharmaceutical substances in the suspension go to both primary and secondary sediments. Among the proposed physicochemical processes, adsorption is the most preferred method for removing pharmaceutical residues [55], which works based on the principle of remediation [3]. The advantages of adsorption are that it allows obtaining high-quality treated wastewater, it is easy and cheap to operate, and it does not result in the production of undesirable by-products [56,57]. It can be used for the treatment of various types of water and wastewater, including those with a high content of organic compounds, which cannot be removed by other methods [58] Adsorption of drugs with the use of porous materials, mainly activated carbon, is known as one of the most effective processes for removing these groups of pharmaceuticals, and is thus widely used. Powdered active carbon is often used in adsorption processes [59,60]. It contains numerous pores of different sizes and has different functional groups on its surface. However, its disadvantage is the difficulty associated with the regeneration of the used adsorbent and the low-selective adsorption of organic adsorbents, especially at low concentrations. Activated charcoal adsorbs a wide spectrum of medicines, especially hydrophobic compounds, owing to its well-developed pore structure, large surface area, and high degree of fragmentation. On the other hand, hydrophilic drugs are inefficiently removed [17,18,61]. A disadvantage encountered with the use of activated charcoal is that the working capacity of the material is significantly reduced if natural organic matter is present, as well as regeneration of the used adsorbent. Regenerative processes significantly affect the pore structure and chemical properties of functional groups in activated carbon, thereby reducing their adsorption efficiency in relation to the removed pharmaceuticals. Thermal regeneration of activated carbon can also cause carbon losses of up to 10% of its mass, which results in the need to purchase new activated carbon. As an alternative, zeolites and mesoporous silica materials can be used. These are characterized by the need for shorter contact time, lower desorption percentage, and better structural stability (which allows regeneration at high temperature) compared with activated carbon, all of which justify their use. This paper presents the general characteristics of zeolites and mesoporous silica materials and an authoritative review of data from research publications, which have not been discussed before in other studies. While individual publications contain results describing the removal efficiency of a selected pharmaceutical (belonging to one of the two groups analyzed), there is no study providing a comparative summary of removal efficiencies and conditions of the experiments conducted for several compounds from a given group and several zeolite sorbents or mesoporous materials. Therefore, efforts have been made to include in this paper the data on the efficiency of zeolites and mesoporous materials to remove the two most common groups of pharmaceuticals—antibiotics and non-steroid pharmaceuticals—from water. The paper reviews the literature on the physicochemical properties of selected zeolites (natural, synthetic, and high silica) and mesoporous silica materials—Mobil Composition of Matter (MCM-41) and Santa Barbara Amorphous (SBA-15)—and their relation to the adsorption of selected antibiotics and non-steroid pharmaceuticals. The zeolites and mesoporous silica materials described in this paper were chosen for this review because of their high availability in the market and their proven effectiveness in removing antibiotics and non-steroidal drugs from aqueous solutions. Zeolites have been shown to have the potential to be successfully used for the adsorption of sulfamethoxazole from water [62]. The adsorption efficiency of zeolites and mesoporous silica materials was characterized taking into account their properties and the diversity of the two analyzed groups of drugs. The paper also discusses the potential possibilities and challenges related to the use of zeolites and mesoporous silica materials in water treatment. The review serves two purposes. Firstly, it allows determining the sorption capacity (described in the literature of zeolites and two mesoporous silica materials) of MCM-41 and SBA-15 in relation to the drugs dissolved in water. Additionally, it can be used to analyze their effectiveness of drug removal and potential use in wastewater treatment and groundwater remediation. Secondly, it allows determining the structural features of the analyzed adsorbent materials, which influence their adsorption of drugs from aqueous solutions. All the collected information may be of help to select materials for water treatment in the future.

## 2. Physicochemical Properties of Zeolites and Mesoporous Silica Materials

### 2.1. Zeolites

The Swedish mineralogist F. Crondtedt used the name zeolite for the first time in 1756. While analyzing the newly discovered mineral, he noticed that it was losing water when heated. In Greek, the word zeolite means “boiling stone” [63]. During the time of their discovery, zeolites were considered as a separate group of minerals [64]. They are defined as tectosilicates, which are inorganic polymers having a three-dimensional structure, and are made up of SiO_4_ tetraeders, some of which can be replaced by AlO_4_ [65,66,67]. A characteristic feature of zeolites is the crystalline structure voids in the form of chambers and channels [68]. The size of zeolites ranges from 3 to 30 Å [69].

Depending on the proportion of silica and aluminium (Si/Al ratio), the properties of zeolites can vary. High-silicon zeolites with a high Si/Al ratio of up to several thousands are produced industrially [70,71]. The hydrophobicity of these zeolites is a beneficial property that facilitates the adsorption of pharmaceuticals from aqueous solutions [72].

The structural features of high-silica zeolites are determined mainly by their framework. A framework type represents the unique channel and frame structure and has the greatest impact on the effectiveness of pharmaceutical adsorption. Mordenite (MOR), faujasite (FAU), and MFI are the type of zeolites selected for this review because they are the most commercially available and have already been tested for the removal of antibiotics and non-steroidal drugs from aqueous solutions. Their structural characteristics are summarized in Table 1.

All the selected framework types are characterized by a large surface area (from 834 to 1211 m^2^ g^−1^) for adsorption. The skeleton density of zeolites is related to their pore volume—zeolites with a lower skeleton density have a larger pore volume [75]. The pore volume of zeolites, which is inversely proportional to skeletal structure density, increases in the following order: FAU > MOR > MFI (Table 1).

Zeolites can also be divided according to their origin into two groups: natural and synthetic. The changes and geological processes taking place in the rocks under hydrothermal conditions favor the formation of natural zeolites. Zeolite deposits occurring in the form of geological deposits, which are profitable for extraction and processing, are found only for some types, such as clinoptilolite, MOR, philipsite, and chabasite. Synthetic zeolites can also be obtained by chemical synthesis. The synthesis of these zeolites is usually carried out under hydrothermal conditions in an alkaline environment [76]. Clay minerals, minerals from the silica group, and by-products of coal combustion (e.g., fly ash) can be used as raw materials for chemical synthesis. The synthesis process changes the chemical and mineral composition and structure of the raw material, consequently giving rise to a zeolite material with new physicochemical properties [77]. Table 2 presents a summary of publications describing the synthesis of selected synthetic zeolites (Na-A, Na-P1, and Na-X) from fly ashes.

The resulting zeolite materials should be filtered, rinsed from NaOH, and dried at about 100 °C for several hours [94]. An advantage of the synthesis of zeolites under laboratory conditions over the natural formation is that the obtained material lasts much shorter [95]. Hence, synthetic zeolites are often used in practice as opposed to natural ones [96]. A cost-effective structural modification is performed before natural zeolites are applied in industries. Moreover, synthetic zeolites are characterized by better texture and adsorption properties compared with natural zeolites. This is because the conditions of the synthesis process can be controlled to obtain zeolite materials with the optimal structure for selected applications. Chemical synthesis of zeolites involves great cost; therefore, the substrates used for synthesis should be cheap mineral or waste materials [97]. Furthermore, zeolites obtained from the conversion of fly ash are characterized by a low production cost, durability, chemical inertia, nonflammability, and developed specific surface area, which are important features found in top-class adsorbents. Another area where zeolites can be applied is to remove pharmaceuticals from water [98,99]. The following subsections present the role and effectiveness of selected zeolites: Zeolite Socony Mobil 5 (ZSM-5); natural Jordanian zeolite (intermediate silica); MOR zeolites with a SiO_2_/Al_2_O_3_ of 18 (MOR18), 200 (MOR200), 240 (MOR240), and 400 (MOR400); modified MOR with an SiO_2_/Al_2_O_3_ ratio of 18 and 240 (TMOR18, TMOR240); magnetic nanoparticles-coated zeolite (MNCZ); zeolite Y; MOR; Slovak natural zeolites from Košice, Slovakia (Zeocem); and FAU-type zeolites (FAU-1, FAU-2). These zeolites were selected thanks to their proven effectiveness in removing antibiotics and non-steroidal drugs from aqueous solutions in studies published to date.

### 2.2. Mesoporous Silica Materials (MCM-41 and SBA-15)

The International Union of Pure and Applied Chemistry (IUPAC) classification defines mesoporous silica materials as porous materials with pores, fissures, cavities, or channels that are deeper than their width [100]. Their pore diameter is in the range of 2–50 nm. According to the IUPAC definition, porous materials also include zeolites, activated carbons, and silica gels. Mesoporous silica materials have gained great interest as they have very small pores that can allow the sorption of large molecules [101,102]. MCM-41 was the first mesoporous silica material to be reported and was described in the Journal of the American Chemical Society and Nature by scientists from the Mobil Research and Development Centre. In turn, in 1998, Zhao et al. synthesized a material that was first described as SBA-15 [103,104,105], which is considered to be the second most popular mesoporous silica material after MCM-41.

Mesoporous silica materials can be classified based on the conditions of synthesis—pH (acidic (e.g., SBA-15) or slightly alkaline (e.g., MCM-41); type of surfactant used (ionic or nonionic), temperature, and amount of water in the synthesis system. The most popular mesoporous silica materials are MCM-41, MCM-48, MCM-50, SBA-15 and SBA-16, KIT-1, FSM-16, and HMS [106,107]. This paper describes the properties of MCM-41 and SBA-15 in detail. These two materials were chosen taking into account their robust design as well as good stability and durability—the properties that make them attractive materials for use as adsorption platforms along mesochannels and allow good adsorption–adsorption interactions.

MCM-41 is a representative of the M41S family of compounds and has an orderly and periodically repeating structure. It is formed from quaternary ammonium salt surfactants. Hexadecyltrimethylammonium bromide (CTAB) is the surfactant most commonly used for synthesis and forms micelles with a positive surface charge. Tetraethoxysilane (TEOS) is usually used as a silica precursor [108]. MCM-41 possesses a hexagonal arrangement of cylindrical pores with a narrow diameter distribution and a large specific surface area. The pore diameter in the range of 1.5–10 nm makes MCM-41 a useful adsorbent for larger particles. Unlike the three-dimensional order found in classical crystals, MCM-41 is characterized by a two-dimensional order. Currently, MCM-41 is still one of the most frequently used types of mesoporous silica nanoparticles in adsorption, catalysis and controlled drug delivery, sensors, and electronics.

SBA-15 is obtained using amphiphilic triblock copolymers with different ratios of ethylene oxides (EOs) to propylene oxides (POs) of the poly EO (PEO)-poly PO (PPO)-PEO or PPO-PEO-PPO type. It is a silica-polymer phase with a hexagonal structure of p6mm symmetry [109]. The pore size of SBA-15 ranges from 5 to 30 nm. The walls of SBA-15 are thicker than those of MCM-41 and range from 3 to 7 nm. Both the pore size and wall thickness of silica can be controlled by appropriate temperature and aging time of the reaction solution [109,110].

The application of mesoporous silica materials is constantly changing. Initially, they were used as molecular sieves with channels wider than zeolites. Their robust construction and long channels make them attractive adsorption materials that allow the diffusion of gases or liquids along the mesocanals and good adsorption–absorption interactions, as mentioned above. Today, mesoporous silica materials have many more potential applications compared with others. They are used to remove pharmaceuticals from water, which is confirmed by the results presented below. It is also possible to modify their properties through different processes (grafting and co-condensation) [111,112,113] or by creating hybrid core/sheath structures, which allows new applications such as molecular printing [114,115].

The widespread occurrence of pharmaceuticals in water indicates the inefficiency of conventional methods used for water and wastewater treatment. Therefore, there is a growing need to implement alternative technologies to optimize the absorption of pharmaceuticals. Among the different methods, adsorption is effective and does not produce unwanted by-products, thus it has been considered promising for removing pharmaceuticals from water and wastewater.

The costs of individual adsorbents depend on many factors (availability, processing requirements, recycling, and duration of use), which is hardly discussed in the literature. Sorbent costs differ based on country and location of use. Costs also vary depending on whether the sorbent is made from by-products of existing industries or is formed from chemically pure raw materials; for example, fly ash-based sorbents are typically produced at low cost. Thus, assessment of the cost of adsorbents is critical to selecting a suitable material for water treatment [116,117,118].

The adsorbent cost analysis should also take into account the factors that influence the determination of the cost of the adsorbent [117,118], including treatment conditions, ease of adsorbate recovery, and reusability of the adsorbent. Pharmaceuticals present in wastewater reduce its mineralization by up to 20%, which increases the cost of treatment [119]. This in turn has an impact on the use of adsorbents. Adsorption is more cost-effective than many other treatment methods [120], and the use of cheaper and sustainable adsorbents derived from waste products would further reduce the expenses.

It can be assumed that the cost of adsorption-based water treatment ranges from $10 to $200 per million liters, depending on the type and amount of adsorbent required [121]. On the other hand, the costs of electrodialysis, electrolysis, ion exchange, and reverse osmosis processes can be as high as $450 per million liters [121]. Increased purification costs in advanced oxidation processes (AOPs) result from the use of chemicals [119] and energy consumption [122]. Adsorption is thus economically more viable. It also has additional advantages including high pharmaceutical removal rates (>90%), low energy consumption, mild operating conditions, and no requirement for by-product addition to the system.

## 3. Factors Affecting the Adsorption of NSAIDs and Antibiotics by Zeolites and Mesoporous Silica Materials

### 3.1. Characteristics of the Selected NSAIDs and Antibiotics

Pharmacokinetic studies have shown that a significant proportion of the administered drugs is excreted in feces and urine [17,123], and thus occurs in domestic sewage. The discharge of expired medication into toilets is another source of wastewater contamination, which unfortunately is difficult to verify and estimate owing to a lack of reliable data. Wastewater discharges from drug production plants also contaminate water with pharmaceuticals [17]. After passing through wastewater treatment plants, pharmaceutical residues end up in surface waters, thereby contributing to the contamination of rivers and streams. The methods currently used for water and wastewater treatment ensure no or low-level purification of water from pharmaceutical residues and/or their metabolites, which results from the lack of appropriate standards and legal regulations forcing the use of appropriate sorption materials that will retain the specific pollutants. It is estimated that about three thousand different pharmaceutical substances are widely used, among which painkillers and antibiotics are the most common, followed by beta-blockers, antidepressants, and hormones [124]. The introduction of pharmaceuticals into the environment is determined by a number of integral factors influencing the pharmacological fate of the drug inside and outside the body. These factors include the degree of consumption of compounds, biotransformation processes, and the behavior of the drug or its metabolite during the wastewater treatment process [19].

NSAIDs are the most commonly used class of painkillers worldwide thanks to their availability over-the-counter (OTC) and frequent recommendations by doctors [125,126]. However, frequent use of NSAIDs has been reported to cause adverse drug reactions. These compounds act by blocking the activity of two isomeric forms of cyclooxygenase, namely COX-1 and COX-2. Cyclooxygenase is an enzyme that catalyzes the synthesis of prostaglandins, thromboxanes, and prostacyclins from arachidonic acid released from cell membrane phospholipids by phospholipases after cell stimulation [48,127,128]. The mechanism of action of NSAIDs was first elucidated by John Vane, who received a Nobel Prize for this research [129,130].

Antibiotics, which are another group of pharmaceuticals commonly found in water and wastewater, are emerging pollutants. They are widely prescribed to treat various types of infections thanks to their bactericidal or bacteriostatic effect. Both NSAIDs and antibiotics are excreted from the human or animal body in an unchanged form, or as biologically active metabolites after biotransformation [131,132]. Therefore, it seems necessary to retain these types of pollutants in wastewater treatment systems, using appropriately developed and selectively functionalized sorption materials such as zeolites or mesoporous silica materials. The characteristics of the selected substances are summarized in Table 3 and Table 4.

### 3.2. Methods of Extraction and Determination of NSAIDs and Antibiotics in the Aquatic Environment

The presence of various pharmaceutical residues in very low concentrations in water and wastewater necessitates the development of sensitive methods for their determination. An extraction technique commonly used to isolate pharmaceuticals is solid-phase extraction (SPE), which enables to determine the concentration of the isolated compounds, especially those present in trace amounts such as NSAIDs and antibiotics. A wide variety of sorbents are used in the SPE process, thus choosing the appropriate filling can significantly influence the recovery of the drugs. A properly selected SPE cartridge allows optimizing the extraction conditions for many analyzed samples. Currently, the sorbents most often used to enrich environmental matrices, for drug analysis of residues, are silica-based or polymer sorbents. Liquid chromatography coupled with mass spectrometry is widely applied for separating pharmaceuticals. Table 5 summarizes the analytical methods used for water and wastewater samples for the determination of the analyzed compounds [148,149,150,151,152,153,154,155,156].

Zeolites are increasingly tested in studies on pharmaceutical residues for determining the preconcentration of aqueous samples in the solid-phase dispersion extraction technique [153,154,155,156]. This technique is characterized by a short experiment time and low reagent consumption, and the use of zeolites as sorbents for pharmaceuticals allows high analyte recovery.

## 4. Potential Applications of Zeolites and Mesoporous Silica Materials in Water Treatment—Discussion

### 4.1. Adsorption of Selected NSAIDs (Sodium Diclofenac, Ibuprofen, and Naproxen) and Selected Antibiotics (Erythromycin and Sulfamethoxazole) on Zeolites

**Figure 3:**
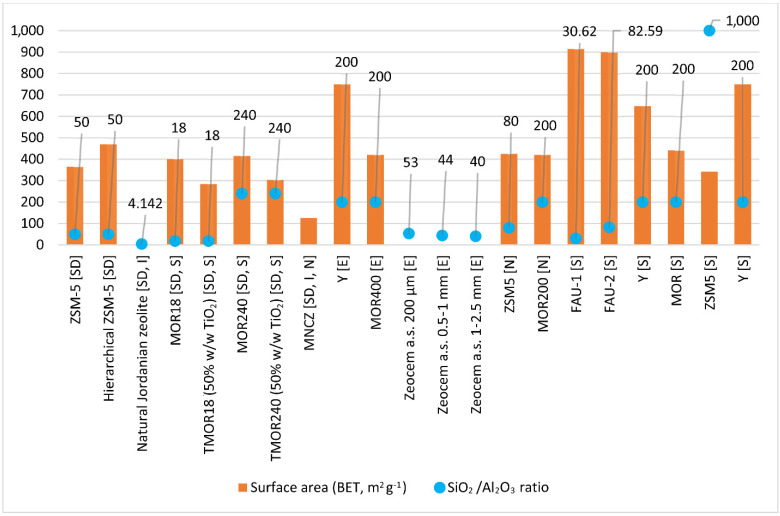
Structural properties of zeolites described in the literature for the removal of selected NSAIDs ([SD]—sodium diclofenac, [I]—ibuprofen, and [N]—naproxen) and selected antibiotics ([E]—erythromycin and [S]—sulfamethoxazole), ZSM-5 [SD], hierarchical ZSM-5 [SD]—[157]; natural Jordanian zeolite [SD, I]—[159] MOR18 [SD, S], TMOR18 (50% (*w*/*w*) TiO_2_) [SD, S], MOR240 [SD, S], TMOR240 (50% (*w*/*w*) TiO_2_) [SD, S]—[162]; MNCZ [SD, I, N]—[163]; Y [E], MOR400 [E]—[98]; Zeocem a.s. 200 μm [E], Zeocem a.s. 0.5–1 mm [E], Zeocem a.s. 1–2.5 mm [E]—[164]; ZSM-5 [N], MOR200 [N]—[99]; FAU-1 [S], FAU-2 [S]—[62]; Y [S], MOR [S], ZSM-5 [S]—[165]; Y [S]—[166].

**Figure 4:**
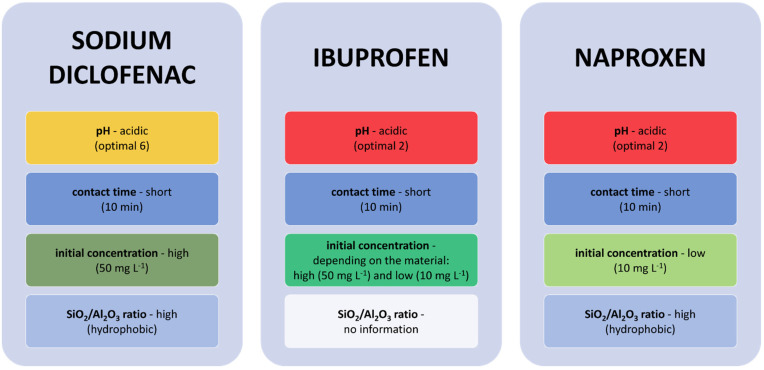
Factors influencing the removal efficiency of selected zeolites: sodium diclofenac—ZSM-5, hierarchical ZSM-5, natural Jordanian zeolite (intermediate silica), TMOR18, TMOR240, MNCZ; ibuprofen—natural Jordanian zeolite (intermediate silica), MNCZ; naproxen—MNCZ, ZSM-5, MOR200.

The improvement of the sorption of diclofenac sodium was influenced by both the geometry of the pores and molecules of the hierarchical zeolite ZSM-5, as well as the interaction with active sites [157]. The BET surface areas of zeolite ZMS-5 and hierarchical zeolite ZMS-5 are shown in Figure 3. In turn, with the use of natural Jordanate zeolite (intermediate silica), it was observed that diclofenac sodium was sorbed best at pH 6 (Figure 4, Table 6). This is because the diclofenac sodium cation can then penetrate the pores of the zeolite. Removal is very fast because, after 10 min, the maximum removal percentage was achieved, similar to MNCZ, in which increasing the contact time was found to have no significant effect on the adsorption of sodium diclofenac [158]. More efficient removal of diclofenac sodium was noted when its initial concentration was higher, because intense interactions occur between the natural zeolite and diclofenac sodium [159]. The use of MOR modified with TiO_2_, which has an SiO_2_/Al_2_O_3_ ratio of 18 and 240 (TMOR18 and TMOR240), made it possible to conclude that adsorption of sodium diclofenac is more effective on zeolites with a higher SiO_2_/Al_2_O_3_ ratio because of the fact that these zeolites are more hydrophobic [160] and have lower negative charges than those with a lower SiO_2_/Al_2_O_3_ ratio [161]. Data on the structural parameters of the starting materials—MOR zeolites with an SiO_2_/Al_2_O_3_ ratio of 18 and 240 (MOR18, MOR240), on which the modifications were made—are presented in Figure 3. On the other hand, sorption of diclofenac sodium on MNCZ confirmed the observations made by Al-rimawi et al. that the removal is more selective in solutions with an acidic pH [158] (Figure 4, Table 6).

Studies on Jordanate natural zeolite showed that ibuprofen is removed effectively when the pH is acidic and the optimal pH is 2 [159]. This result was also confirmed when MNCZ was used as an adsorbent [158]. The contact time may be short, and 10 min is sufficient for effective removal (Figure 4, Table 6). If the initial concentration is high, resistance to mass transfer of pharmaceutical molecules between the aqueous and solid phases of Jordanate natural zeolite is quickly overcome [159]. On the other hand, for MNCZ, the efficiency of ibuprofen removal decreases at higher concentrations and improves at lower concentrations [158].

ZSM-5 and MOR zeolites having an SiO_2_/Al_2_O_3_ ratio of 200 (MOR200), which were used to remove naproxen, were in a powdered form. Data on the SiO_2_/Al_2_O_3_ ratios presented in Figure 3 show that MOR200 zeolite is more hydrophobic compared with ZSM-5 zeolite. It was confirmed that lower sorption of naproxen occurs at alkaline pH, which may be caused by the increase in the amount of hydroxyl ions and formation of water complexes delaying sorption (Figure 4, Table 6). Naproxen sorption occurs quickly, and the contact time required for removal is only 10 min [158]. Removal occurs better with zeolites having a higher SiO_2_/Al_2_O_3_ ratio [99] and a low initial concentration [158].

Three zeolites were used to remove erythromycin, including two synthetic ones (MOR zeolites with an SiO_2_/Al_2_O_3_ ratio of 400—MOR400 and Y zeolite) and natural Slovak zeolite from Zeocem a.s. with different fractions (200 μm, 0.5–1 mm, and 1–2.5 mm). The characteristics of MOR400 and zeolite Y are presented in Figure 3. Zeolite turned out to be a better synthetic sorbent, which was proved by TG curve and XRD analyses. The analyses showed that adsorption of erythromycin occurred in the entire zeolite structure, as well as in micropores [98]. Erythromycin removal from wastewater (concentrations: 16.0 ng L^−1^—Stupava treatment plant; 37.0 ng L^−1^—Devínska Nová Ves treatment plant) was tested using natural zeolite Zeocem a.s. with three fractions. A 30 min contact time resulted in over 90% removal at both concentrations for the finer fraction. Such high efficiency was achieved because the pH condition was optimal (the pH of the wastewater must be lower than the pKa of erythromycin, i.e., 8.88) [164] (Table 6). Studies investigating the adsorption efficiency of zeolite Y were carried out using water collected at the outlet of a wastewater treatment plant in Ferrara (northern Italy), where the actual concentration of erythromycin was 1.10 µg g^−1^, and the results confirmed that 100% removal was achieved with this zeolite [98].

**Figure 5:**
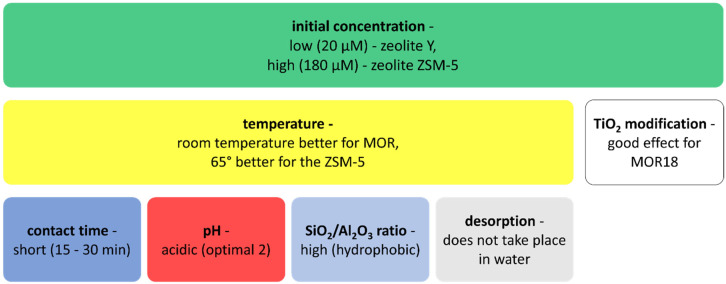
Factors influencing the sorption of sulfamethoxazole on zeolites: FAU-1, FAU-2, zeolite Y, MOR, ZSM-5, TMOR18, and TMOR240.

The contact time needed to remove sulfamethoxazole is 15–30 min, and the optimal pH is 2 (Table 6). For the pH found in the wastewater (i.e., 7.25), an elimination efficiency of 43% was achieved [62]. The removal of sulfamethoxazoles is effective on high-silicon zeolites [166]. The characteristics of zeolites are presented in Figure 3. Studies carried out using MOR and ZSM-5 zeolites proved that temperature influences the effectiveness of sorption. Sorption with MOR was more effective at RT, while better results for ZSM-5 were achieved at the temperature of 65 °C. Research also showed that the efficiency of sorption on individual zeolites is influenced by the initial concentration; for low concentrations (20 µM) of contaminants, it is best to use zeolite Y, while for high concentrations (180 µM), ZSM-5 can work better. A positive aspect of using zeolite Y, MOR, and ZSM-5 is the fact that sorption is irreversible [165]. Of these sorbents, zeolite Y turned out to be the best. Modification of MOR18 zeolite with TiO_2_ significantly increased the adsorption of sulfamethoxazole [162] (Figure 5).

Al-Rimawi and colleagues (2019) studied the removal of sodium diclofenac and ibuprofen using natural zeolite Jordanate (intermediate silica). They determined that the optimum pH for ibuprofen is 2 and for sodium diclofenac is 6. Their study showed that, based on the chemical structure of zeolites, at a low ibuprofen concentration, the drug will interact with zeolite through a relatively strong interaction of the carboxylic group and oxygen atoms combined with silicon and aluminium elements. At pH 6, sodium diclofenac may participate in strong interactions with zeolite molecules through the following mechanism: sodium cation may penetrate the pores of the zeolite and participate in electrostatic interactions between the cation and amine group of diclofenac. Removal of sodium diclofenac on the zeolite may be related to the fact that, at higher values, the proton concentration (H^+^) is reduced, and thus its competition with sodium cations for binding to diclofenac is minimal, which results in stronger interactions between the drug and the zeolite (drug amine and sodium cation are in the zeolite pores).

It was observed that, in the case of both diclofenac and ibuprofen, the maximum removal percentage was reached after 10 min, but the optimum contact time was taken as 80 min to ensure appropriate contact. The maximum removal of sodium diclofenac was estimated at 2.0 g L^−1^ and of ibuprofen at 1.0 g L^−1^. The concentrations of both pharmaceuticals were tested in the range of 10–50 mg L^−1^. The ability to remove both medicines increased with the increasing concentration. This effect can be explained as follows: a higher initial concentration increases the driving force, which allows overcoming the resistance to mass transfer of pharmaceutical molecules between the aqueous and solid phases. Moreover, the increase in absorption capacity with increasing starting concentration may also result from a more intense interaction between the natural zeolite and medicines. The authors determined the adsorption capacity of the tested zeolite for sodium diclofenac at 4.8 mg g^−1^ according to the Langmuir isotherm and for ibuprofen at 1.23 mg g^−1^ according to the Freundlich isotherm [159].

Modifications of MOR with an SiO_2_/Al_2_O_3_ ratio of 18 and 240 (coated with “Kronoclean 7000” (Kronos, Germany) and TiO_2_ powder, designated as modified MOR, with an SiO_2_/Al_2_O_3_ ratio of 18 and 240 (TMOR18 and TMOR240), were carried out to remove pharmaceuticals such as diclofenac and sulfamethoxazole. Modification with TiO_2_ powder caused the specific surface area (BET) to decrease, but allowed the formation of new mesopores and macropores. The effects of surface reduction and micropore formation of TMOR18 and TMOR240 zeolites did not have a significant impact on the adsorption of medicines. However, the SiO_2_/Al_2_O_3_ ratio in the structure of zeolites influenced the adsorption of pharmaceuticals because zeolites with a higher SiO_2_/Al_2_O_3_ ratio are more hydrophobic and have lower negative charges compared with those with lower SiO_2_/Al_2_O_3_ ratios. On TMOR240, diclofenac was removed only by 20–30%. Diclofenac has a negative charge, which can cause electrostatic repulsion between MOR240 and the drug. However, the negative surface charge of TMOR240 was assumed to be quite low. Modification with TiO_2_ zeolite MOR240 and MOR18 showed almost an insignificant influence on the adsorption of the tested medicines, except for the adsorption of sulfamethoxazole on TMOR18. On the other hand, modification of MOR18 zeolite with TiO_2_ significantly increased the adsorption of sulfamethoxazole because the surface was negatively charged [162].

Various sorption parameters, such as contact time, solution pH, and initial concentration (diclofenac sodium, ibuprofen, and naproxen), were investigated to optimize the reaction conditions for magnetic nanoparticles coated zeolite (MNCZ). The pH of the solution affects the removal efficiency of the tested pharmaceuticals. However, it has been proved that, as the pH changes from acidic to alkaline conditions, the tested compounds are less efficiently removed on MNCZ. Lower sorption efficiency was observed at an alkaline pH, which may be due to an increase in the amount of hydroxyl ions and the formation of aqueous complexes, delaying sorption. Consequently, the values of adsorption observed for a solution with a pH of 2.0 were 99.58% for diclofenac sodium, 98.75% for ibuprofen, and 99.79% for naproxen, while the values determined for a solution with pH 11.0 were 93.99%, 90.79%, and 90.69%, respectively. Although pH was an important factor influencing sorption on MNCZ, it was proved that this material had a high ability to remove tested medicines over a wide pH range (2–9). This information is very important for the future use of MNCZ for drinking water and/or wastewater treatment. The time of contact with MNCZ had no significant effect on the adsorption of diclofenac sodium, and MNCZ showed a high potential to adsorb ibuprofen and naproxen. The removal efficiency was over 95% after just 10 min. The starting concentration had no significant effect on the removal efficiency of the tested pharmaceutical at low concentrations. Nevertheless, a significant reduction in the removal efficiency of the tested compounds, especially ibuprofen, was observed with an increase in its concentration [158].

### 4.2. Adsorption of Selected NSAIDs (Diclofenac, Ibuprofen, and Ketoprofen) and Selected Antibiotics (Sulfamethoxazole, Tetracycline, and Trimethoprim) on MCM-41 and SBA-15

Inorganic mesoporous silica materials such as SBA-15 and MCM-41 are alternative adsorbents used for water treatment [55]. They are characterized by high porosity, uniform and narrow pore sizes, ordered arrangement of pore structures, large pore volume, and large surface area, which increases their adsorption capacity. Langmuir and Freundlich isothermal parameters that determine the adsorption of pharmaceuticals from aqueous solutions are listed in Table 7 for selected MCM-41 and SBA-15 adsorbents and selected NSAIDs and antibiotics. These silica-based porous materials have already been used to remove pharmaceutical residues by other researchers [167].

**Figure 6:**
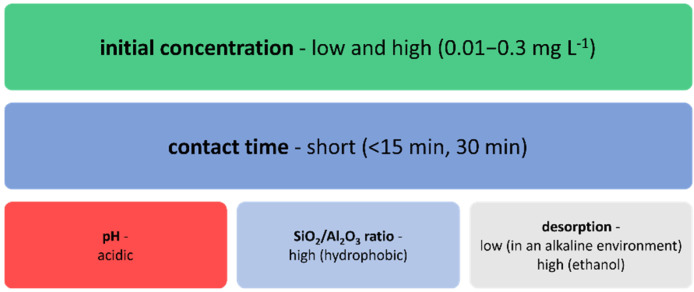
Factors influencing the sorption of diclofenac, ibuprofen, and ketoprofen on mesoporous materials: SBA-15, MCM-41, and TMS-SBA-15.

SBA-15 material was used in two independent studies for the removal of diclofenac, ibuprofen, and ketoprofen (Figure 6). The contact time needed to remove these three NSAIDs was <15 min [168], while two other studies showed that the required contact time was 30 min [55,169]. This can be because of the ordered mesoporous structure of the materials used in the research. SBA-15 showed better adsorption efficiency compared with MCM-41. The adsorption process was more effective at low pH, which proves that the interaction occurring between drugs and the surface of mesoporous silica is hydrophilic [55,169]. Desorption of these pharmaceuticals in an alkaline environment was low, which indicates that they were strongly adsorbed to SBA-15, MCM-41, and TMS-SBA-15, whereas, with the use of ethanol, the desorption was found to be high [55,168,169].

**Figure 7:**
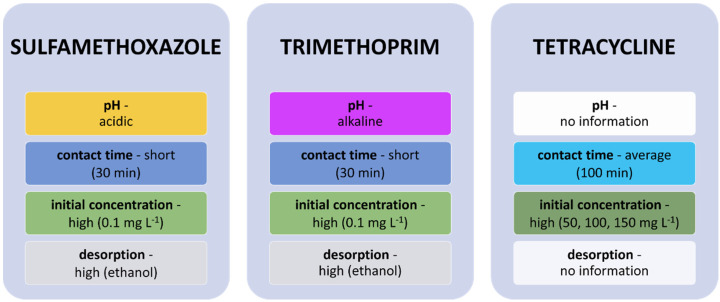
Factors influencing the sorption of sulfamethoxazole and trimethoprim on TMS-SBA-15 and tetracycline on A-MCM-41.

Sulfamethoxazole and trimethoprim were removed on TMS-SBA-15 material (Figure 7). The initial concentration of both of these antibiotics was high, yet adsorption occurred after a short contact time of 30 min. Complete desorption of these antibiotics was achieved with the use of ethanol. One difference was noted in the effect of pH on the adsorption of these compounds. Sulfamethoxazole is an anionic compound; therefore, it was removed efficiently in low-pH solutions. On the other hand, trimethoprim is a cationic compound, and an increase in the pH of the solution made its sorption more effective [169]. A-MCM-41—mesoporous material MCM-41 impregnated with zeolite A—was used to remove tetracycline in a study. Three high concentrations of the antibiotic were tested, and the results proved that tetracycline adsorption was most effective at the highest concentration. The removal process took 100 min (Figure 7) and, after this time, tetracycline adsorption was very slow, as most of the reactive sites were occupied [170].

SBA-15 is a promising adsorbent for the removal of diclofenac, ibuprofen, and ketoprofen not only from surface water, but also from wastewater from the pharmaceutical industry, which have high concentrations of these pharmaceuticals. For SBA-15 (2.0 g L^−1^), the removal efficiency of diclofenac, ibuprofen, and ketoprofen was determined to be 66.7%, 95.1%, and 91.2%, respectively. In the dose range of 0.1–1.0 g L^−1^ (SBA-15), the adsorption of diclofenac, ibuprofen, and ketoprofen increased almost linearly with an increase in the concentration of the adsorbent, but the increase in the adsorbent dose from 1.0 to 2.0 g L^−1^ did not have a significant effect. Adsorption of all medicines (diclofenac, ibuprofen, and ketoprofen) reached equilibrium in a very short time (<15 min). This may be owing to the ordered mesoporous structure of SBA-15. The strongly pH-dependent adsorption of drugs suggests that the interaction between the drugs and the mesoporous silica surface is hydrophilic. Moreover, the low percentage of desorption of pharmaceuticals from the silica surface in alkaline media suggests that they are strongly adsorbed to SBA-15. After sorption, SBA-15 can be regenerated by combustion without any material losses, owing to the stability of the mesoporous silica structure at temperatures up to 850 °C [55].

## 5. Summary and Perspectives

Pharmaceuticals, which have long been present in the environment for 2–3 decades, are an environmental concern. They have been detected by various studies, and their dangerous effects on flora, fauna, and humans have been noticed [41,44,171,172,173]. Pharmacological contaminants are not completely removed in sewage treatment plants, which causes their migration to surface waters. For this reason, there is a constant search for advanced and effective processes for removing these pollutants. Adsorption and AOPs are processes that can be used to effectively remove medicines from water and wastewater. However, a disadvantage of AOPs is that they generate many oxidation and transformation by-products, some of which are toxic. Adsorption is advantageous over AOP, as it does not result in the formation of new products. However, it requires a large amount of adsorbent, which has consequences related to the sorbent itself, such as the need for regeneration or disposal after use, as well as consequences related to the adsorbed pharmaceuticals and their derivatives, which must be eliminated. Zeolites and mesoporous silica materials can be cheap adsorbents because they are both produced from fly ashes that are considered waste. The removal of NSAIDs and antibiotics using adsorption is currently applied on a laboratory scale or in small-scale implementation studies. After sorption, materials can often be used to improve soil properties or can be regenerated by burning without damaging their structure while destroying the adsorbed medicines. Table 5 and Table 6 present the examples of zeolites and mesoporous silica materials used to remove selected NSAIDs (diclofenac, ibuprofen, ketoprofen, and naproxen) and selected antibiotics (erythromycin, sulfamethoxazole, tetracycline, and trimethoprim) from aqueous solutions. They also provide information on adsorption kinetics and the applied test conditions. Finally, this review is complemented by a commentary covering research on drugs in the environment. Pharmaceutical disposal is currently a growing and active area of research as new medicines are launched every year and their environmental impact is significant. The following points should thus be noted:

Wastewater treatment plants must adopt new technologies for the effective removal of pharmaceuticals.

The removal technologies should be affordable and easy to apply rapidly on a large scale at a low cost.

Standards defining the maximum allowable concentration should be implemented to reduce the amount of pharmaceuticals entering the environment (aquatic ecosystems) from wastewater treatment plants.

Advanced methods for accurate and continuous detection of drugs should be developed and applied, especially in rapidly developing industrial countries such as India and China.

Continuous research should be conducted on environmental systems to determine how pharmaceuticals affect flora, fauna, and microorganisms.

NSAIDs and antibiotics are two of the most frequently recommended pharmaceuticals and are essential in many cases for pain relief or to treat bacterial infections. These compounds are excreted from the human body either in an unchanged form or as metabolites after biotransformation, which unfortunately leads to their persistence in the environment and poses potential risks to the ecosystem. Therefore, it is necessary to investigate their sorption and disposal at the stage of wastewater treatment. It is also extremely important to develop an efficient and inexpensive technology for retaining these compounds on appropriate sorption beds as well as to legally regulate the need to introduce a stage for their retention in the wastewater treatment process. The development and implementation of a sorption procedure, based on zeolites or mesoporus materials, for modern NSAIDs and antibiotics may give rise to a wastewater treatment system that can also be used for other frequently used drugs such as hormones, antidepressants, anticancer substances, or beta-blockers.

Thus, this paper provides a thorough literature review on zeolites and mesoporous silica materials used to retain selected medicines. This review may be helpful to carry out further experimental studies aimed at creating an appropriate sorption bed for effectively retaining drugs. The review focused mainly on pharmaceuticals that are commonly used, but are chemically different in terms of structure and particle size. The sorption bed prepared using this approach will be universal and will enable retaining a wide range of undesirable chemical compounds in wastewater.

On the basis of the conducted analysis, future research should be carried out in terms of the use of the described materials as sorbents for pharmaceuticals from wastewater (and not only from solutions prepared in the laboratory). The materials used in the described research had a dusty form, which makes it difficult to use them in column flow experiments. For this reason, their granulation should be included in further considerations. The granulation process opens up many research opportunities related to, for example, the selection of appropriate consolidation processes. Subsequently, it is possible to study the effect of effective modification of the sorbent surface in order to increase the simultaneous co-sorption of cationic and anionic chemical compounds. It is also worth considering the methods of regeneration after sorption and re-use of materials in the same sorption process without losing sorption capacity, efficiency, and selectivity. Another issue that may be addressed in the future is the profitability of using modified sorbents or sorbents after regeneration. The cost of obtaining sorbents should be estimated and compared with materials generally available on the market.

## Figures and Tables

**Table 1:** Key properties of three commonly used frameworks of zeolites.

Frame-Work Type	Ring Number and Pore Opening Size [73]		Framework Density [73]	Accessible Area Maximum [74]	Maximum Diameter of a Sphere [74]
	(Å × Å)	(Å × Å)	(T-Atoms per 1000 Å)	(m^2^ g^−1^)	(Å)
FAU	12 ring 7.4 × 7.4	-	12.7	1211.42	11.24
MOR	12 ring 6.5 × 7.4	8 ring 2.6 × 5.7	17.2	1010.22	6.70
MFI	10 ring 5.1 × 5.5	10 ring 5.3 × 5.6	17.9	834.41	6.36

**Table 2:** Selected publications on the synthesis of synthetic zeolites Na-A, Na-P1, and Na-X from fly ashes.

Type of Zeolite	Conditions of Synthesis			NaOH/Fly Ash Ratio	Reference
	NaOH [M]	T [°C]	t [h]		
Na-A	0.5–3.5	60	10–48	0.5–3.5	[78]
	2.0	100	2	0.8	[79]
	2.2	85	12	0.23	[80]
	2.0–5.0	100–150	0.5–6	0.5–1.6	[81]
Na-P1	2.8–5.0	25	48	0.28–0.5	[82]
	2.0	90–150	12	-	[83]
	3.0	103	12	0.5	[84]
	1.0–3.0	90	21	0.4–1.2	[85]
	0.5–5.0	150–200	3–48	-	[86]
	3.0	125	8	-	[87]
	0.4–0.5	120	3–24	0.08–0.64	[88]
	3.0	125	9	0.96	[86]
	1.16	80–320	6	0.28	[89]
	1.0	105	24	0.8	[90]
	-	100	12–48	1.0	[91]
Na-X	3.0	90	24–72	0.3	[92]
	3.0	75	24	2.4	[90]
	-	10	120	-	[93]
	3.0	75	24	0.33	[65]

**Table 3:** Characteristics of the discussed NSAIDs.

Common Name	Diclofenac	Ibuprofen	Ketoprofen	Naproxen
CAS Number	15307-86-5	15687-27-1	22071-15-4	22204-53-1
Molecular weight	296.15	206.28	254.28	230.26
pKa ^a^	4.15	4.91	4.45	4.15
log Kow ^a^	4.51	3.97	3.12	3.18
CEC ^b^ (ng L^−1^)	4560	194,711	48,978	827,999
Classification group	acetic acid derivatives	propionic acid derivatives	propionic acid derivatives	propionic acid derivatives
Therapeutic use/mechanism of action	NSAID/ non-selective COX inhibitor	NSAID/ non-selective COX inhibitor	NSAID/ non-selective COX inhibitor	NSAID/ non-selective COX inhibitor
Half-lives, hours	2	1.2–2	1.1–4	12–17
Metabolite	hydroxy metabolites, glucuronic acid, sulfate, and taurine	hydroxylated and carboxylated derivatives	glucuronide metabolite	desmethylnaproxen, glucuronide metabolit
Reference	[133]	[134,135]	[136]	[137,138]

**Table 4:** Characteristics of the discussed antibiotics.

Common Name	Erythromycin	Sulphamethoxazole	Tetracycline	Trimethoprim
CAS Number	114-07-8	723-46-6	60-54-8	738-70-5
Molecular weight	733.93	253.28	444.44	290.32
pKa ^a^	8.88	1.6 5.7	3.30	7.12
log Kow ^a^	3.06	0.89	1.37	0.91
CEC ^b^ (ng L^−1^)	-	9.8 × 10^7^	6.7 × 10^7^	3.3 × 10^6^
Classification group	macrolide antibiotic	sulfonamides	tetracyclines	anisoles
Therapeutic Use/mechanism of action	Antibiotic/ bacteriostatic	Antibiotic/ bacteriostatic	Antibiotic/ bacteriostatic	Antibiotic/ bactericidal
Half-lives, hours	2–3.5	10	6–12	8–10
Metabolite	N-desmethylerythromycin	hydroxysulfamethoxazole, acetylsulfamethoxazole ulfamethoxazole N4-hydroxylamine, sulfamethoxazole N-glucuronide	not metabolized	demethylated 3′- and 4′-metabolite
Reference	[139,140]	[141,142]	[143]	[142,144]

^a^ Data were adopted from other sources [145,146]; ^b^ CEC values were adopted from [147]; ^a^ pKa —dissociation constant, ^a^ log Kow —octanol−water partition coefficient, CEC—critical environmental concentration.

**Table 5:** Methods used for the extraction and determination of the analyzed compounds.

Common Name	Extraction Technique/Sorbent	Determination Method	Level (µg L^−1^)	Reference
Diclofenac	SPE/polymer	LC-MS/MS	2.0–6.30 0.91–1.90 0.18–2.60	[148]
	SPE/hydrophilic-lipophilic polymer	LC-MS	0.116	[149]
	SPE/hydrophilic-lipophilic polymer	LC-MS	0.113–4.882	[150]
Ibuprofen	SPE/ hydrophilic-lipophilic polymer	LC-MS/MS	27.30	[151]
Ketoprofen	SPE/ hydrophilic-lipophilic polymer	LC-MS	0.031–3.511	[150]
Naproxen	SPE/ hydrophilic-lipophilic polymer	LC-MS	22.50	[149]
	SPE/ hydrophilic-lipophilic polymer	LC-MS/MS	19.90	[151]
Erythromycin	SPE/ hydrophilic-lipophilic polymer	LC-MS	0.509–0.149	[152]
	SPE/ hydrophilic-lipophilic polymer	LC-MS/MS	0.785	[153]
Sulphamethoxazole	SPE/polymer	LC-MS	0.376–0.572	[154]
	SPE/ hydrophilic-lipophilic polymer	LC-MS	2.060	[149]
	SPE/ hydrophilic-lipophilic polymer	LC-MS/MS	0.024	[153]
Tetracycline	SPE /hydrophilic-lipophilic polymer	LC-MS	146.0	[155]
Trimethoprim	SPE /hydrophilic-lipophilic polymer	LC-MS/MS	0.007	[156]
	SPE/ polymer	LC-MS	0.27–0.94	[154]
	SPE /hydrophilic-lipophilic polymer	LC-MS	1.140	[149]

**Table 6:** Parameters of zeolites for the removal of selected NSAIDs (diclofenac, ibuprofen, and naproxen) and selected antibiotics (erythromycin, sulfamethoxazole, tetracycline, and trimethoprim).

Adsorbate	Framework Type of Zeolite (Si/Al Ratio)	Dose (g L^−1^)	Contact Time (h)	T (°C)	Concentrations	pH	Reference
Sodium diclofenac	zeolite ZSM-5 hierarchical ZSM-5	0.05	1.0	29.85	10–1000 µM	-	[157]
Sodium diclofenac	natural Jordanian zeolite (intermediate silica)	2.0	1.2	-	10.0; 20.0; 40.0; 50.0 × 10^4^ µg L^−1^	6.0	[159]
Sodium diclofenac	TMOR18 (50% *w*/*w* TiO_2_) TMOR240 (50% *w*/*w* TiO_2_)	0.7	-	-	100 μg L^−1^	-	[162]
Sodium diclofenac	MNCZ	0.05	-	30 ± 1	100 µg L^−1^	2.0–9.0	[158]
Erythromycin	zeolite Y	-	-	-	0–5 × 10^3^ µg L^−1^	-	[98]
Erythromycin	Zeocem a.s. 200 μm Zeocem a.s. 0.5–1 mm Zeocem a.s. 1–2.5 mm	0.05	0.5	-	0.016 µg L^−1^ 0.037 µg L^−1^	pH = 6.85 pH = 7.01	[164]
Ibuprofen	natural Jordanian zeolite (Intermediate silica)	1.0	1.2	-	10.0; 20.0; 40.0; 50.0 × 10^4^ µg L^−1^	2.0	[159]
Ibuprofen Naproxen	MNCZ	0.05	-	30 ± 1	100 µg L^−1^	2.0–9.0	[158]
Naproxen	ZSM5 MOR200	0.05	-	-	2 µg L^−1^	6.0	[99]
Sulfametoksazole	FAU-1 FAU-2	0.50	2.0	-	1.0 × 10^5^ μg L^−1^	6.5	[62]
Sulfamethoxazole	zeolite Y MOR ZSM5	0.50	24.0	21 21 65	30 µM	-	[165]
Sulphamethoxazole	TMOR18 (50% *w*/*w* TiO_2_) TMOR240 (50% *w*/*w* TiO_2_)	0.70	-	-	100 μg L^−1^	-	[162]
Sulphamethoxazole	zeolite Y	0.50	1.0	-	50 µM	5–8	[166]

**Table 7:** Comparative evaluation of adsorbent capacities for the removal of selected pharmaceuticals from aqueous solutions.

Adsorbent	Adsorbate	pH	Temp (°C)	Conc. Range (mg L^−1^)	Surface Area (m^2^ g^−1^)	Freundlich Sorption Capacity (KF)	Langmuir Sorption Capacity (mg g^−1^)	Reference
MCM-41	Diclofenac	7.0	25	0.04−0.3	755	0.05	0.11	[168]
SBA-15	Diclofenac	7.0	25	0.04−0.3	890	0.11	0.13	[168]
SBA-15	Diclofenac	5.0	25	0.01−0.3	737	0.72	0.34	[55]
SBA-15	Ibuprofen	5.0	25	0.01−0.3	737	1.50	0.41	[55]
SBA-15	Ketoprofen	5.0	25	0.01−0.3	737	1.09	0.28	[55]
AMCM-41	Tetracycline	7.0	30	300	485	368.58	415.10	[170]
AMCM-41	Tetracycline	7.0	40	300	485	364.21	417.50	[170]
AMCM-41	Tetracycline	7.0	50	300	485	362.15	419.30	[170]
